# An assessment of out of pocket payments in public sector health facilities under the free maternal healthcare policy in Ghana

**DOI:** 10.1186/s13561-023-00423-0

**Published:** 2023-01-28

**Authors:** Maxwell Tii Kumbeni, Agani Afaya, Paschal Awingura Apanga

**Affiliations:** 1 Department of Health Management and Policy, College of Public Health and Human Sciences, Oregon State University, Corvallis, USA; 2 Mo Im Kim Nursing Research Institute, College of Nursing, Yonsei University, Seoul, South Korea; 3 Department of Nursing, School of Nursing and Midwifery, University of Health and Allied Sciences, Ho, Ghana; 4 Kentucky Department for Public Health, Frankfort, USA

**Keywords:** Free maternal healthcare policy, National health insurance scheme, Out of pocket payment, Skilled delivery services, Public sector health facilities, Health system factors, Ghana

## Abstract

**Background:**

The free maternal healthcare policy was introduced in Ghana in 2008 under the national health insurance scheme as a social intervention to improve access to maternal health services. This study investigated the prevalence of out of pocket (OOP) payment among pregnant women with valid national health insurance who sought skilled delivery services at public sector health facilities in Ghana. The study also assessed the health system factors associated with OOP payment.

**Methods:**

We used data from the Ghana Maternal Health Survey (GMHS), which was conducted in 2017. The study comprised 7681 women who delivered at a public sector health facility and had valid national health insurance at the time of delivery. We used multivariable logistic regression analysis to assess factors associated with OOP payment, whiles accounting for clustering, stratification, and sampling weights.

**Results:**

The prevalence of OOP payment for skilled delivery services was 19.0%. After adjustment at multivariable level, hospital delivery services (adjusted Odds Ratio [aOR] = 1.23, 95% Confidence Interval [CI] = 1.00, 1.52), caesarean section (aOR = 1.73, 95% CI = 1.36, 2.20), and receiving intravenous infusion during delivery (aOR = 1.31, 95% CI = 1.08, 1.60) were associated with higher odds of OOP payment. Women who were discharged home 2 to 7 days after delivery had 19% lower odds of OOP payment compared to those who were discharged within 24 hours after delivery.

**Conclusion:**

This study provides evidence of high prevalence of OOP payment among women who had skilled delivery services in public sector health facilities although such women had valid national health insurance. Government may need to institute measures to reduce OOP payment in public sector facilities especially at the hospitals and for women undergoing caesarean sections.

## Background

The free maternal healthcare policy in Ghana was rolled out in 2008 under the national health insurance scheme [1]. This was part of the Government of Ghana’s social interventions to increase access to maternal healthcare services in the country. The national health insurance scheme is being funded by premiums, the national health insurance tax, social security and national insurance trust, government funding, and returns from investments [2, 3]. Since the inception of this policy, Ghana has had a significant improvement in maternal health service utilization such as antenatal care, skilled delivery, and postnatal care services [4–6]. The increase in utilization of maternal health services has consequently contributed to reduction in under-5 and maternal mortalities [7, 8].

Under the free maternal healthcare policy, pregnant women are exempted from paying premiums for registration or annual renewal for the national health insurance scheme [1]. Once the pregnancy is confirmed by a doctor/midwife/nurse, they are enrolled onto the national health insurance scheme without a fee to access maternal health services [1, 9]. The range of services covered under this program include antenatal care contacts, essential medicines, and essential laboratory services. It also covers skilled delivery services, including caesarean sections and postnatal services for the mother and newborn for up to 3 months [9, 10].

Pregnant women can access the free maternal health services from both the public and private sector health facilities with a valid national health insurance card provided such facilities are accredited by the national health insurance authority [11]. However, the public sector health facilities provide majority of the maternal health services in Ghana [12]. According to the recent Ghana Maternal Health Survey, public sector health facilities provide 85% of the skilled delivery services in Ghana [13]. Therefore, public sector health facilities play a critical role to the success of the free maternal healthcare services if they adhere to the policy guidelines of the national health insurance scheme [14, 15].

The Government of Ghana is solely responsible for managing the national health insurance scheme and has had some challenges, including their inability to timely reimburse funds to health facilities [15–17]. This has compelled some health facilities, including public sector health facilities to introduce OOP payments to pregnant women seeking maternal healthcare services [16, 18, 19]. The introduction of OOP payments for maternal health service utilization has the potential to defeat the purpose of the free maternal healthcare policy and possibly derail the gains made by Ghana towards improving maternal and child health outcomes [13]. This is because OOP payments are associated with low utilization of maternal health services, including skilled delivery services [20, 21].

Studies in Ghana on OOP payments have focused on both public and private sector health facilities [18, 19, 22], but to the best of our knowledge none has assessed OOP payments in only public sector health facilities. It is therefore important to assess the prevalence of OOP payment in public sector facilities as these facilities are the bedrock of the free maternal healthcare policy. Furthermore, studies on OOP payments have not been nationally representative and may not be generalizable to the Ghanaian population [18, 22, 23]. There is also dearth of knowledge on health system factors that might be associated with OOP payments in public sector health facilities. Therefore, the aims of this study were 1) to assess the prevalence of OOP payment among pregnant women with a valid national health insurance who sought skilled delivery services in public sector health facilities and 2) to assess health system factors associated with OOP payment. These findings will be useful for policy makers to take steps towards improving maternal health utilization and avert any decline in the gains Ghana has made in improving maternal and child health outcomes.

## Methods

### Data sources

Data for the study was extracted from the Ghana Maternal Health Survey (GMHS), which was conducted in 2017. The survey was conducted by Ghana Statistical Service (GSS) with technical support from Inner City Fund (ICF) through the Demographic and Health Survey (DHS) program. GMHS used a multi-stage sampling where the first stage involved the selection of enumeration areas with probability proportional to the sizes of enumeration areas. In the second stage, households were selected from each enumeration area using systematic random sampling. Details of the sampling procedure is publicly available [24]. The 2017 GMHS was conducted among women aged 15–49 years who delivered a live birth or stillbirth from the period between 2012 to 2017.

### Study population

Our study population were women aged 15–49 years who delivered at a public sector health facility in Ghana and had valid national health insurance at the time of delivery. Women who delivered at a private sector health facility or at home were excluded from the study. Pregnant women who had a private health insurance or did not have any health insurance at the time of delivery were also excluded from the study. Our total sample size was 7681 women.

### Variable selection

#### Outcome variable

Our outcome variable of interest was OOP payment from a mother with a valid health insurance card during skilled delivery  at public sector health facility. A valid health insurance was an active health insurance with the national health insurance scheme at the time of receiving skilled delivery service. The outcome variable was generated out of three forms of OOP payment; payment to see a doctor/midwife/nurse, payment for laboratory services and payment for medicines. Pregnant women who paid for anyone of these three were categorized as OOP payment, otherwise they were categorized as not having made OOP payment. The outcome was coded as a dummy variable “1 for yes and 0 for no”.

#### Independent variables

The primary independent variables of interest were type of health facility, forceps or vacuum delivery, blood transfusion during delivery, intravenous infusion during delivery, delivery by caesarean section and length of stay after delivery. Type of health facility was categorized as (hospital, health center/clinic/Community-based Health Planning and Services (CHPS) compound); forceps or vacuum delivery (yes, no); blood transfusion during delivery (yes, no); intravenous infusion during delivery (yes, no); delivery by caesarean section (yes, no), and length of stay after delivery (24 hours, 2 to 7 days and more than a week). The secondary independent variables of interest were place of residence (urban, rural); age category (15–19, 20–34, 35–49); parity (primiparous, multiparous); education (no formal education, primary education, secondary education, higher education) and wealth (poor, middle, rich). The variable selection was based on literature review [19], and their availability in the GMHS dataset [13].

### Data analysis

Data analysis was conducted using Stata/SE 14.0 (Stata Corp LLC, College Station, Texas USA). Descriptive statistics was used to assess the prevalence of OOP payment and characteristics of the study population. We conducted bivariate analysis using logistic regression to assess the relationship between independent variables and OOP payment. A statistical significance of *p*-value < 0.05 was set for inclusion of independent variables into the multivariable logistic regression model. Adjusted odds ratios (aORs) at 95% confidence interval (CI) were estimated. We accounted for clustering, stratification, and sampling weights in all our analysis because of the complex sampling design.

## Results

### Characteristics of study participants

The prevalence of OOP payment was 19.0%. Approximately, 70.0% of the women delivered at the hospital, 3.8% delivered through forceps or vacuum extraction and 16.1% delivered by a caesarean section. More than half (57.3%) received intravenous infusion during delivery, 5.4% had blood transfusion and 46.0% were discharged home within 24 hours after delivery. More than half (53.3%) of the women were resident in an urban area, 27.2% were primiparous, 56.0% had secondary education, and more than a third (36.1%) were in the poor wealth category (Table 1).

**Table 1:** Characteristics of study participants (*n* = 7681)

Variables	Frequency (%)
Out of pocket payment	
Yes	1459 (19.0)
No	6222 (81.0)
Type of health facility	
Hospital	5323 (69.3)
Health center/clinic/CHPS	2358 (30.7)
Forceps or vacuum delivery	
Yes	292 (3.8)
No	7386 (96.2)
Blood transfusion during delivery	
Yes	415 (5.4)
No	7263 (94.6)
Intravenous infusion during delivery	
Yes	4399 (57.3)
No	3279 (42.7)
Length of stay after delivery^a^	
24 hours	3533 (46.0)
Two to seven days	3779 (49.2)
More than a week	369 (4.8)
Delivery by caesarean section	
Yes	1236 (16.1)
No	6442 (83.9)
Place of residence	
Urban	4094 (53.3)
Rural	3587 (46.7)
Age Category in years	
15–19	384 (5.0)
20–34	5054 (65.8)
35–49	2243 (29.2)
Parity	
Primiparous	2097 (27.3)
Multiparous	5584 (72.7)
Education	
No formal education	1475 (19.2)
Primary education	1390 (18.1)
Secondary education	4301 (56.0)
Higher education	515 (6.7)
Wealth	
Poor	2773 (36.1)
Middle	1674 (21.8)
Rich	3234 (42.1)

^a^Length of stay after delivery (women who stayed for more than 24 hours but less than 48 hours were classified under two days)

### Factors associated with OOP payment

Women who delivered at the hospital were 1.23 times as likely to make OOP payment compared to those who delivered at a health center/clinic/CHPS compound (aOR = 1.23, 95% Confidence Interval [CI] = 1.00, 1.52). Compared with women who did not receive intravenous infusion during delivery, those who received intravenous infusion were 1.31 times as likely to make OOP payment during skilled delivery services (aOR = 1.31, 95% CI = 1.08, 1.60). Women who were delivered by caesarean section had 1.73 times the odds of OOP payment compared to those who had vaginal delivery (aOR = 1.73, 95% CI = 1.36, 2.20). Women who were resident in urban areas (aOR = 1.28, 95% CI = 1.04, 1.59), had primary education (aOR = 1.47, 95% CI = 1.11, 1.93) or had secondary education (aOR = 1.63, 95% CI = 1.30, 2.10) also had higher odds of OOP payment during skilled delivery services. Duration of stay after delivery for 2 to 7 days was associated with 19% lower odds of OOP payment compared to women who were discharged within 24 hours after delivery (aOR = 0.81, 95% CI = 0.67, 0.97) (Table 2).

**Table 2:** Factors associated with out of pocket payment during skilled delivery services

Variables	Unadjusted OR (95% CI)	Adjusted OR (95% CI)
Type of health facility		
Health center/clinic/CHPS	1.00	1.00
Hospital	1.55 (1.28, 1.87)**	1.23 (1.00, 1.52)*
Intravenous infusion during delivery		
No	1.00	1.00
Yes	1.61 (1.36, 1.92)**	1.31 (1.08, 1.60)**
Length of stay after delivery		
24 hours	1.00	1.00
Two to seven	1.06 (0.91, 1.26)	0.81 (0.67, 0.97)*
More than a week	1.62 (1.15, 2.30)*	0.97 (0.66, 1.42)
Delivery by caesarean section		
No	1.00	1.00
Yes	2.00 (1.66, 2.42)**	1.73 (1.36, 2.20)**
Place of residence		
Rural	1.00	1.00
Urban	1.55 (1.30, 1.86)**	1.28 (1.04, 1.59)*
Education		
No formal education	1.00	1.00
Primary education	1.63 (1.25, 2.14)**	1.47 (1.11, 1.93)**
Secondary education	1.96 (1.59, 2.41)**	1.63 (1.30, 2.10)**
Higher education	1.89 (1.30, 2.76)**	1.29 (0.85, 1.95)
Wealth		
Poor	1.00	1.00
Middle	1.30 (1.06, 1.60)*	1.03 (0.81, 1.29)
Rich	1.66 (1.40, 2.00)**	1.14 (0.89, 1.45)

*OR* Odds ratio, *CI* Confidence Interval, 95% confidence intervals in brackets; ^*^ *p* < 0.05, ^**^ *p* < 0.01, 1 = Reference category

## Discussion

Our study investigated the prevalence of OOP payment and its associated health system factors among pregnant women with valid national health insurance who sought skilled delivery services in public sector health facilities in Ghana. We found that one out of every five pregnant women made OOP payment during skilled delivery. Pregnant women who delivered at the hospital, delivered by caesarean section, and received intravenous infusion during delivery had higher odds of OOP payment. Women who were discharged home after 2 to 7 days after delivery had lower odds of OOP payment during skilled delivery services compared to those who were discharged home within 24 hours after delivery.

The Ghana national health insurance scheme comprehensively covers all maternal health services, including antenatal care, delivery services and postnatal care in public sector health facilities [9, 10]. However, our study found that about one-fifth of the women paid OOP to either see a practitioner (i.e., doctor/midwife/nurse), for medicines or laboratory service at a public healthcare facility. These OOP payments subtly introduced at some public sector health facilities may be a way to keep providing healthcare services amidst the lack of timely reimbursement of national health insurance funds from government [15–17]. This is a major challenge for health facilities as the main source of funding for health facilities is funds from the national health insurance reimbursement [25]. The lack of timely reimbursement of funds pose a threat to pregnant women having to pay OOP for skilled delivery services, which ideally should be free because the clients had a valid health insurance card [16, 18, 19]. These OOP payments have the potential to cause a decline in utilization of maternal health services, particularly skilled delivery services [26]. This may undermine the progress Ghana has made towards improving maternal and child health outcomes.

OOP payment was more prevalent among women who delivered at the hospital compared to those who delivered at other health facilities (i.e., health center/clinic/CHPS compounds). A similar finding has been reported in Burkina Faso [27]. A possible explanation for this finding is that hospitals have a higher prevalence of skilled delivery services, including referrals from health centers/clinics/CHPS compounds [24]. Furthermore, some of the medications used at the hospital level may not be covered under the national health insurance scheme essential medicines list [28]. The implication of this finding is that pregnant women who are referred to the hospital from other health facilities (i.e., health center/clinic/CHPS compounds) may be reluctant to honor such referrals because of the higher cost of OOP payment. This has the potential to reduce skilled delivery services in the hospital as well as maternal and child health outcomes [20, 21, 26].

We also found that women who delivered by caesarean section had higher odds of OOP payments compared to those who delivered per vagina. A number of reasons could have accounted for this finding. Some pregnant women may have opted to undergo elective caesarean section [29], which require them to pay for the service although it should be free under the free maternal healthcare policy [30]. Additionally, pregnant women who undergo caesarean section may likely use epidural anaesthesia for pain management [31], and some of these medicines are not included in the essential medicines list of the national health insurance scheme [28, 30]. This finding may also explain why our earlier finding on OOP payment being higher at the hospital because caesarean sections are mostly performed at the hospital level in Ghana.

Women who received intravenous infusion treatment during delivery also had higher odds of OOP payment compared to those who did not receive intravenous infusion. Intravenous infusions are mostly indicated as fluid replacement or as medicines to manage skilled delivery [32–34]. The use of intravenous infusions are more prevalent in hospitals, where OOP payments are also more common. We also found that women who were discharged home after 2 to 7 days after delivery had reduced odds of OOP payment compared to those who were discharged within 24 hours. This finding is surprising; however, a possible explanation could be that mothers who were unable to afford OOP cost stayed longer in the health facility until such debts were waived by the facility before they were discharged home.

Women living in urban areas and women who had primary or secondary education were more likely to incur OOP cost during skilled delivery services compared to women living in rural areas and women who had no formal education. Similar findings has been reported elsewhere [27]. Hospitals are predominantly found in urban areas in Ghana [35], and so pregnant women in urban areas are more likely to utilize hospitals during skilled delivery services, which tend to be associated with OOP payment compared to those in rural areas, where health centers/clinics/CHPS compounds are predominant. Educational attainment is associated with women living in urban areas [36, 37], and are therefore more likely to utilize hospital services. Moreover, educated women are more likely to be economically stable and may opt for elective caesarean section thereby incurring OOP cost for the services [30].

Our study had some limitations and strengths. We cannot infer causality because of the cross-sectional nature of the data. Furthermore, most of our variables were self-reported and recall bias could have been introduced. We could also not account for variables outside of the GMHS dataset (e.g., supply related factors). However, our study reports the national prevalence of OOP payment among pregnant women with valid national health insurance in public sector health facilities in Ghana. It is also the first study to assess the health system factors associated with OOP payments. Furthermore, the use nationally representative data makes our findings generalizable to Ghana.

## Conclusion

One out of every five pregnant women incurred OOP payment for skilled delivery services in public sector health facilities despite having a valid national health insurance. Skilled delivery services at the hospital, caesarean section delivery and receiving intravenous infusion during delivery were associated with OOP payment. The high prevalence of OOP payment is a potential barrier to accessing skilled delivery services in Ghana. Government may need to institute measures to reduce OOP payment in public sector facilities especially at hospitals and for caesarean sections.

## Data Availability

Data is available upon request.

## Abbreviations

OOP: Out of Pocket
DHS: Demographic and Health Survey
GMHS: Ghana Maternal and Health Survey
CHPS: Community-based Health Planning and Services
